# Differential Effects of Phosphatase Inhibitors on the Calcium Homeostasis and Migration of HaCaT Keratinocytes

**DOI:** 10.1371/journal.pone.0061507

**Published:** 2013-04-30

**Authors:** Olga Ruzsnavszky, Beatrix Dienes, Tamás Oláh, János Vincze, Tamás Gáll, Enikő Balogh, Gábor Nagy, Róbert Bátori, Beáta Lontay, Ferenc Erdődi, Laszlo Csernoch

**Affiliations:** 1 Department of Physiology, University of Debrecen, Debrecen, Hungary; 2 Department of Medical Chemistry, University of Debrecen, Debrecen, Hungary; 3 Department of Microbial Biotechnology and Cell Biology, University of Debrecen, Debrecen, Hungary; 4 1st Department of Internal Medicine, University of Debrecen, Debrecen, Hungary; Brigham & Women's Hospital - Harvard Medical School, United States of America

## Abstract

Changes in intracellular calcium concentration ([Ca^2+^]_i_) as well as in the phosphorylation state of proteins have been implicated in keratinocyte wound healing revealed in scratch assays. Scratching confluent HaCaT monolayers decreased the number of cells displaying repetitive Ca^2+^ oscillations as well as the frequency of their Ca^2+^-transients in cells close to the wounded area and initiated migration of the cells into the wound bed. In contrast, calyculin-A (CLA) and okadaic acid (OA), known cell permeable inhibitors of protein phosphatase-1 and 2A, increased the level of resting [Ca^2+^]_i_ and suppressed cell migration and wound healing of HaCaT cells. Furthermore, neither CLA nor OA influenced how scratching affected Ca^2+^ oscillations. It is assumed that changes in and alterations of the phosphorylation level of Ca^2+^-transport and contractile proteins upon phosphatase inhibition mediates cell migration and wound healing.

## Introduction

In mammalian cells changes in intracellular calcium concentration ([Ca^2+^]_i_) control a wide variety of functions, including proliferation, secretion, motility and contractility [1]. Rapid Ca^2+^ transients are required for fast cellular processes, like synaptic transmission and muscle contraction, while slower Ca^2+^ responses – as repetitive Ca^2+^ transients and waves – are responsible for gene transcription and cell proliferation. Calcium ions underlying Ca^2+^ oscillations are released from the endoplasmic reticulum (ER) via inositol 1,4,5-trisphosphate receptors (IP_3_R) and ryanodine receptors (RyR), and often spread through the cytoplasm as a regenerative Ca^2+^ wave [2]. This phenomenon is well-known in excitable cells, but some non-excitable cells, such as endothelial cells [3], osteoblasts [4], and chondrocytes [5] were also shown to display calcium oscillations.

Activity of the Ca^2+^ release channels responsible for Ca^2+^ oscillations can be increased or decreased depending on their phosphorylation state. The serine/threonine protein phosphatases 1 and 2A (PP1 and PP2A) have been found to co-purify with protein kinase A (PKA) and IP_3_R, which is reminiscent of their interaction with RyR2 in heart muscle. The presence of PP1 and PP2A ensures a tight regulation of the phosphorylation status of the receptor and, therefore, its activity [2]. The ability of PP1 to dephosphorylate RyR was demonstrated in both skeletal and cardiac muscle [6], which could indicate that a similar complex exists not only in heart muscle, but in other cell types as well, with the involvement of RyR1 and/or IP_3_R.

Several inhibitors were used to study the role of protein phosphatases. Calyculin A (CLA) inhibits the activity of both PP1 and PP2A with similar effectiveness in *in vitro* assays, while okadaic acid (OA) reduces PP2A activity with higher efficiency than that of PP1. Neither calyculin A nor okadaic acid inhibit acid or alkaline phosphatases or phosphotyrosine protein phosphatases [7], [8].

Albeit protein kinase and phosphatase enzymes together with the changes in [Ca^2+^]_i_ have been implicated to possess a significant role in the regulation of cell migration their interaction has not been studied in wound healing. During wound healing, keratinocytes initiate migration from the wound edge by extending lamellipodia into a fibronectin-rich provisional matrix, which was enhanced by protein-serine/threonine kinase inhibitors [9]. In contrast, okadaic acid which can increase the phosphorylation level of myosin II, together with an increased stress fiber formation was shown to decrease hepatic cell migration [10]. On human primary keratinocytes, when epidermal growth factor receptors were activated and the phosphorylation of extracellular signal-related kinase (ERK) was increased cell migration and wound healing was enhanced. Similarly, during β2 adrenergic receptor stimulation, when PP2A was activated and ERK was dephosphorylated, the extent of cell migration was decreased. On the other hand, inhibition of PP2A by 10 nM okadaic acid resulted in an increased extent of migration [11].

In fish keratinocytes migration can be stopped with a burst increase of [Ca^2+^]_i_ [12] and it was suggested that the endogenous Ca^2+^-transients occurring during Ca-oscillations may exert a resensitization-desensitization control during substrate guided movements of keratinocytes. Similar results were obtained with human primary keratinocytes where the Ca^2+^ uptake via nicotinic acetilcholine (Ach) receptors caused the decrease of the migratory distance of the cells [13]. Phosphatase inhibitors as OA and CLA were shown to potentiate the thapsigargin-induced elevation in [Ca^2+^]_i_ in human neutrophils [14], although it was not evident whether these effects were due to their phosphatase inhibitory action. Arachidonic acid (AA) -induced Ca^2+^ release and entry was enhanced by both CLA and tautomycin (TM) in parotid acini, while OA had no influence on the release but inhibited entry [15]. Similarly, CLA augmented twitch Ca^2+^-transients and cell shortenings in both control and isoproterenol-treated cardiac myocytes [16]. The above findings imply that the changes in [Ca^2+^]_i_ and phosphorylation of key proteins (by activating kinases or inhibiting phosphatases) may be interrelated and their combined effect might mediate cell migration.

In our experiments migration and proliferation of HaCaT keratinocytes in the presence and absence of phosphatase inhibitors (CLA and OA) were tested following a scratch of confluent cells. Spontaneous Ca^2+^ oscillations were observed and analyzed in unscratched cells and cells next to the scratch. In the latter increased resting [Ca^2+^]_i_ and decreased oscillations could be observed. Phosphatase inhibitors increased both the extent of spontaneous Ca^2+^ oscillations and the resting [Ca^2+^]_i_, while they decreased migration of the cells. These results suggest that protein phosphatases and oscillatory changes in [Ca^2+^]_i_ have distinct roles in keratinocyte migration.

## Materials and Methods

### 1. Culturing HaCaT keratinocytes

HaCaT keratinocytes [17] were cultured in Dulbecco's modified Eagle's medium (DMEM, Sigma, Budapest, Hungary) supplemented with 2 mmol/L L-glutamine, 10% fetal calf serum (Sigma) and antibiotics (50 NE/ml penicillin, 50 µg/ml streptomycin and 1.25 µg/ml fungizone) at 37°C temperature in a 5% CO_2_ atmosphere as described in our previous reports [18].

### 2. Measuring the intracellular calcium concentration

Resting [Ca^2+^]_i_ was monitored using Fura-2, as described in our earlier reports [19]. In brief, cells were placed on a glass coverslip and loaded with 10 µM Fura-2 AM (acetoxymethyl ester) for 60 minutes. Cells were then equilibrated in Tyrode's solution (in mmol/L, 137 NaCl, 5.4 KCl, 0.5 MgCl_2_, 1.8 CaCl_2_, 11.8 Hepes-NaOH, 1 g/L glucose, pH 7.4, all from Sigma) for 30 min at room temperature. Cells were incubated with CLA (10 nM) for 1 hour, or with OA (50 nM) for 2 hours before the measurements, and during the experiments cells were bathed continuously in these phosphatase inhibitors. The scratch was made with a sterile 10 µl pipette tip in the confluent culture. Coverslips with Fura-2-loaded cells were placed on the stage of an inverted fluorescent microscope (Diaphot; Nikon, Tokyo, Japan). The excitation wavelength was changed between 340 and 380 nm by a microcomputer-controlled dual-wavelength monochromator (Deltascan; Photon Technology International, New Brunswick, NJ), whereas the emission was monitored at 510 nm using a photomultiplier at 10 Hz acquisition rate of the ratios. [Ca^2+^]_i_ was calculated from the ratio of fluorescence intensities (R = F340/F380) using an in vivo calibration (R_min_ = 0.2045, R_max_ = 8.315, K_d_ β = 1183) as described [20]. All measurements were performed at room temperature.

### 3. Confocal measurements

Calcium transients were monitored with LSM 5 Live confocal laser scanning microscope (Zeiss, Oberkochen, Germany). The protocol described earlier [21] was followed. In brief, cells were incubated with 10 µM Fluo-4-AM for 30 min at 37°C. Calcium imaging was performed in normal Tyrode's solution, and Tyrode's solution supplemented with the phosphatase inhibitors CLA (10 nM) or OA (50 nM).

Two dimensional (x–y) images were used to monitor the fluorescence intensity of the cultures. Series of x-y images were recorded using a 40× oil immersion objective with 2 frames/second scan speed. The resolution was 512×512 pixels. Fluo-4 was excited with an argon ion laser. All measurements were performed at room temperature.

### 4. Data analysis and statistics

Series of x-y images were analyzed using ImageJ and a custom-developed computer program. All cells were manually marked as region of interest (ROI) in case of each image series in ImageJ. Time-series data containing the mean fluorescence values for each ROI on each frame were exported for further analysis by the custom-developed program.

As the first step of the analysis normalized fluorescence values (*F_n_ = F/F_0_*) were calculated. For each ROI *F_0_* values were determined as the mean of fluorescence values not corresponding to an event (not exceeding the mean fluorescence for the given ROI across the frames by more than 40% of the standard deviation of the same values). The normalized time-series data were analyzed independently for each ROI.

One-dimensional stationary wavelet transform was applied as described by Szabó et al. [22]. In brief, this transformation separates the original signal into higher and lower frequency components in an optimal way. These components are called wavelet levels: *W_i_* denotes the *i*-th level; lower indexed levels correspond to higher frequency components. The first wavelet level, containing almost exclusively high-frequency noise, was removed from the signal. The low-pass filtered signal after the 8^th^ of the wavelet transform was used as a background signal and all normalized values were divided by this background signal before further analysis in order to remove slow changes of fluorescence level in the ROI not caused by the studied spontaneous transients.

The 4^th^ and 5^th^ wavelet levels were used to detect events. An event was identified in a ROI on the given frame if at least one of the corresponding *W_4_* and *W_5_* values was higher than a preset threshold (0.01). Contagious regions of such points were extended to the nearest local minima on *F_n_* and these regions were considered events for further analysis.

### 5. Time-lapse photography

An inverse microscope was equipped with a high sensitivity video camera, and was connected to a custom-built image acquisition computer system as described earlier [23]. Custom-designed illumination was developed to minimize heat- and foto-toxicity. Operation of the spectrally warm-white light emitting diodes was synchronized with image acquisition periods. Cell cultures on glass coverslips, in Petri dishes were placed on the microscope. Photographs were taken in every minute. The time of the exposure was indicated in each frame. Snapshots were converted into video films by speeding up the projection to 30 exposures/s. Individual photographs were selected as they are shown in the figures. The measurements were performed at 37°C in a 5% CO_2_ atmosphere.

### 6. Cell migration assay

Cell migration was measured by using fibronectin-coated (FN) Quantitative Cell Migration Assay following the procedure provided by the manufacturer (Merck Millipore, Budapest, Hungary). Briefly, HaCaT cells were starved 18 hours prior passage in serum free DMEM medium. 2.5×10^5^ cells were plated on BSA (as control) and FN-coated membrane in Boyden Chamber. DMEM culture medium completed with 20% FBS was applied in the lower chamber. Cells were incubated in the absence or in the presence of 50 nM OA and 10 nM CLA for 18 hours. Migration of the cells to the other side of the membrane was assessed by measuring the optical density of dye infiltered cells at 540 nm in a plate reader.

### 7. Statistical methods

Averages are presented as mean ± standard error of the mean. Statistical analysis was performed using SigmaStat (Aspire Software International, Ashburn, VA, USA). Most of the observed data failed to show normal distribution (p<0.05), therefore nonparametric tests were used to assess the significance of differences. Significance of differences between non-scratched and scratched groups was assessed by the Mann-Whitney U test. Kruskal-Wallis One Way Analysis of Variance on Ranks was carried out to determine whether there were significant differences between more than two groups and subsequently an appropriate post-hoc test was used for pairwise comparisons. Ratios resulting from repeated measurements on the same coverslips showed normal distribution and were therefore analyzed using the parametric One Way Analysis of Variance. Differences were regarded significant at p<0.05.

## Results

In our experiments spontaneous calcium transients of HaCaT keratinocytes were observed. The changes in calcium oscillations upon physical damage to neighbouring cells (i. e. scratch) followed by wound healing were investigated. The effects of two different phosphatase inhibitors (CLA and OA) were also studied on the wound healing and calcium homeostasis of the cultures.

### 1. Characterization of spontaneous calcium oscillations of HaCaT keratinocytes

Series of x-y images were recorded both on Fluo-4 loaded control and drug treated cultures, then these cultures were scratched with a pipette tip and cells next to the wound were examined in the same way. 500 images with 500 ms intervals were taken from 3–6 different visual fields of each culture before and after scratching. ROIs were selected on every individual cell, and the number of oscillating cells and characteristics of the oscillations were determined with a custom-developed computer program.

**Figure 1:**
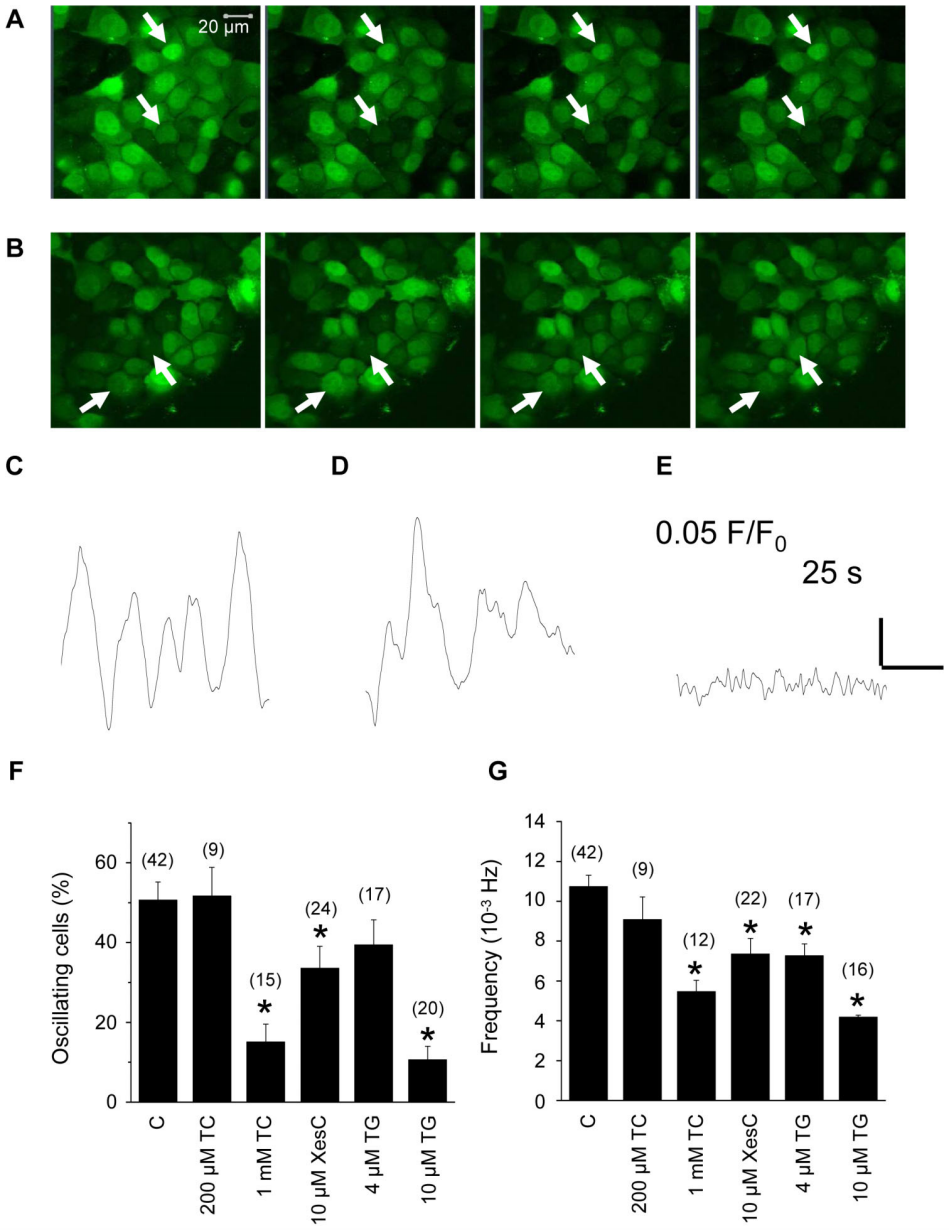
Spontaneous Ca^2+^ oscillations of keratinocyte cultures. Series of confocal x-y images showing the spontaneous Ca^2+^ oscillations of a representative keratinocyte culture without scratch (**A**) and a culture after scratch (**B**). Arrows show examples of oscillating cells. Calibration was the same in all images. Original magnification was 40×. Representative curves showing the fluorescence intensity of a single cell from a non-scratched culture (**C**) and from a cell next to the scratch (**D**). Cultures were imaged by confocal microscopy, sampling rate was 2 Hz. For reference, panel **E** represents the time course of fluorescence ratio of a cell with no spontaneous oscillations. Percentage of oscillating cells (**F**) and average frequency of oscillations (**G**) under control conditions and in the presence of 200 µM or 1 mM tetracaine, 10 µM Xestospongin C, and 4 or 10 µM thapsigargin.

Confluent HaCaT keratinocyte cultures displayed slow and repetitive spontaneous [Ca^2+^]_i_ transients as observed by confocal laser scanning microscopy. Figure 1 displays images of an unscratched (Fig. 1A) and scratched (Fig. 1B) area from the HaCaT keratinocyte monolayer with arrows pointing to cells presenting elevations in [Ca^2+^]_i_ (calcium oscillations) at different timepoints. Note that not only cells marked by arrows but others as well presented clear changes in fluorescence intensity.

Figure 1C, D present the time course of fluorescence change in representative cells from non-scratched (Fig. 1C) and scratched (Fig. 1D) cultures. Note the slow waves representing oscillatory changes in [Ca^2+^]_i_. Similar observations were made in case of the application of CLA and OA. For comparison, Figure 1E shows the time course of fluorescence intensity of a non-oscillating cell. In all cases fluorescence was expressed as normalised to resting fluorescence (F_0_).

To assess the underlying mechanisms responsible for the Ca^2+^ oscillations we tested a number of chemical agents known to either block the IP_3_R (large concentration of tetracaine, low concentration of Xestospongin C) or RyR (low concentration of tetracaine) and the Ca^2+^ATPase of the endoplasmic reticulum (thapsigargin). Figure 1F and G show, respectively, the percentage of oscillating cells and the frequency of oscillations under control conditions and in the presence of 200 µM or 1 mM tetracaine, 10 µM Xestospongin C, and 4 or 10 µM thapsigargin.

Tetracaine in the concentration assumed to affect only RyR neither influenced the percentage of oscillating cells nor the frequency of oscillations. On the other hand, both Xestospongin C, and tetracaine in 1 mM, where it is known to inhibit the IP_3_R as well, significantly reduced both parameters. Similar observation was made with 10 µM thapsigargin. These results indicate that IP_3_R mediated Ca^2+^ release plays an important role in the generation of spontaneous oscillations of HaCaT keratinocytes.

#### 1.1. The rate of oscillation of scratched cultures

**Figure 2:**
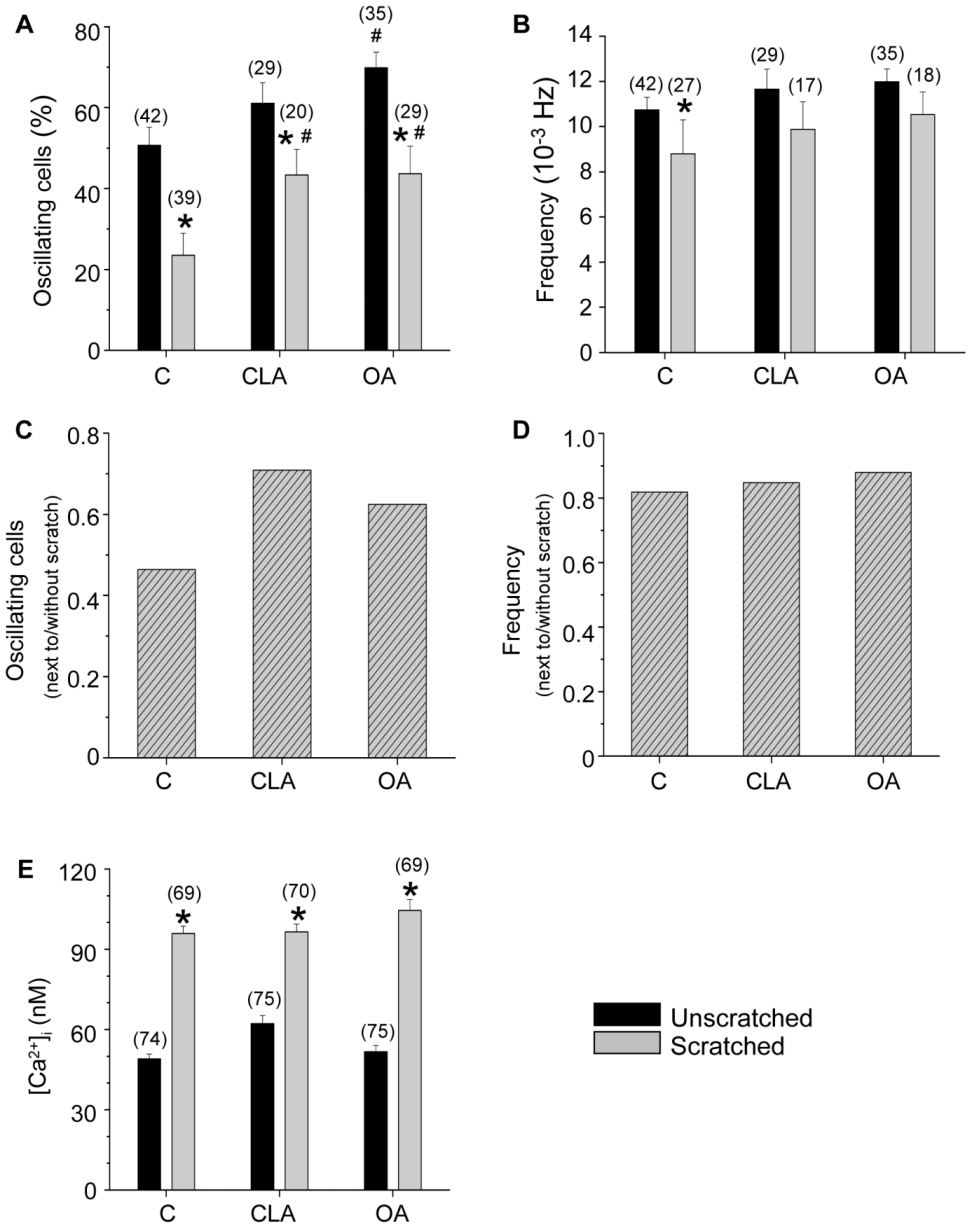
Effect of phosphatase inhibitors on the spontaneous calcium oscillations and [Ca^2+^]_i_ of the keratinocytes. Percentage of oscillating cells in control, CLA treated, and OA treated cultures (**A**). Average frequency of calcium oscillations in those cells where such oscillations could be observed (**B**). Percentage of oscillating cells (**C**) and the frequency of oscillations (**D**) calculated as a ratio from corresponding values obtained from cells next to the scratch and in cells from non-scratched areas. Resting [Ca^2+^]_i_ of control, CLA treated, and OA treated cells (**E**). Numbers in parentheses give either the number of cells (**E**) or the number of fields of view (**A, B,**) measured. (C: control, CLA: calyculin A, OA: okadaic acid). * p<0.05, unscratched *vs* scratched, # p<0.05, control *vs* phosphatase inhibitor.

In control cultures the percentage of oscillating cells (Fig. 2A) decreased significantly (*p*<0.05) in scratched cultures near the scratch as compared to before scratching (50.7±4.5%, n = 42, in unscratched cultures and 23.5±5.4%, n = 39, near the scratch; n = the number of fields of view). In case of CLA or OA treated cultures this significant difference was also observable between the corresponding scratched and unscratched cultures (61.1±5.1%, n = 29, in unscratched cultures and 43.3±6.4%, n = 20, near the scratch; 69.9±3.8%, n = 35, in unscratched cultures and 43.7±6.9%, n = 29, near the scratch, respectively). In other words, cells near the scratch were less likely to display calcium oscillations independent of the presence or absence of phosphatase inhibitors.

Note, on the other hand, that the ratio of oscillating cells was significantly higher in cells treated with phosphatase inhibitors both before and after the scratch. This was true for all cases except for the unscratched control *vs* unscratched CLA treated cultures, where, albeit the tendency was present, the significance was not reached. These observations clearly suggest that while neither CLA nor OA influenced the effect of scratching, they both interfered with the basic mechanism responsible for the generation of the spontaneous increases in [Ca^2+^]_i_.

Not only the percentage of the oscillating cells but also the frequency of calcium oscillations (Fig. 2B) was significantly lower in the scratched control cells (8.8±1.5×10^−3^ Hz, n = 27) than in the unscratched control (10.7±0.6×10^−3^ Hz, n = 42) (*p*<0.05). The presence of the phosphatase inhibitors did not alter significantly the frequency of oscillations as measured before and after the scratch was made (11.7±0.9×10^−3^ Hz, n = 29 in unscratched, and 9.9±1.2×10^−3^ Hz, n = 17 in scratched, CLA treated; and 12.0±0.6×10^−3^ Hz, n = 35 in unscratched, and 10.5±1.0×10^−3^ Hz, n = 18 in scratched, OA treated cells). Nevertheless the same tendency was present in untreated and in CLA and OA treated cells, namely, the frequency of calcium transients was less in cells next to the scratch as compared to cells from unscratched cultures. Furthermore, the presence of phosphatase inhibitors also raised the frequency of oscillations in cells from both unscratched and scratched cultures as compared to control cells, although these differences were not significant (Fig. 2B). For better comparison the percentage of the oscillating cells and the frequency of oscillations for the cells next to the scratch are also shown after normalized to the values obtained for cells from non-scratched areas (Fig. 2C and 2D, respectively). The ratio of cells showing Ca^2+^-oscillations next to the scratch was higher in the presence of phosphatase inhibitors than under control conditions.

#### 1.2. Effect of phosphatase inhibitors and scratching on the resting [Ca^2+^]_i_

Resting [Ca^2+^]_i_ was measured in Fura-2 loaded control and phosphatase inhibitor treated cells (Fig. 2E; 49±2 nM, n = 74 in unscratched control, 62±3 nM, n = 75 in CLA treated, and 52±2 nM, n = 75 in OA treated cells). After scratching the resting [Ca^2+^]_i_ of the cells near the scratch was significantly higher in all three cases as compared to unscratched cultures (96±3 nM, n = 69 in scratched control, 97±3 nM, n = 70 in CLA treated, and 104±4 nM, n = 69 in OA treated cells).

#### 1.3. Characterisation of the Ca^2+^ transients

Two basic characteristics, the *amplitude* and the *Full Time at Half Maximum (FTHM)* of the individual spontaneous Ca^2+^ transients were examined. The former was calculated as the change in fluorescence intensity (*ΔF/F_0_*) and is a measure of the change in [Ca^2+^]_i_, while the latter was determined as the duration time of the transients at the half of the amplitude, and correlates with the duration of the Ca^2+^ transients.

**Figure 3:**
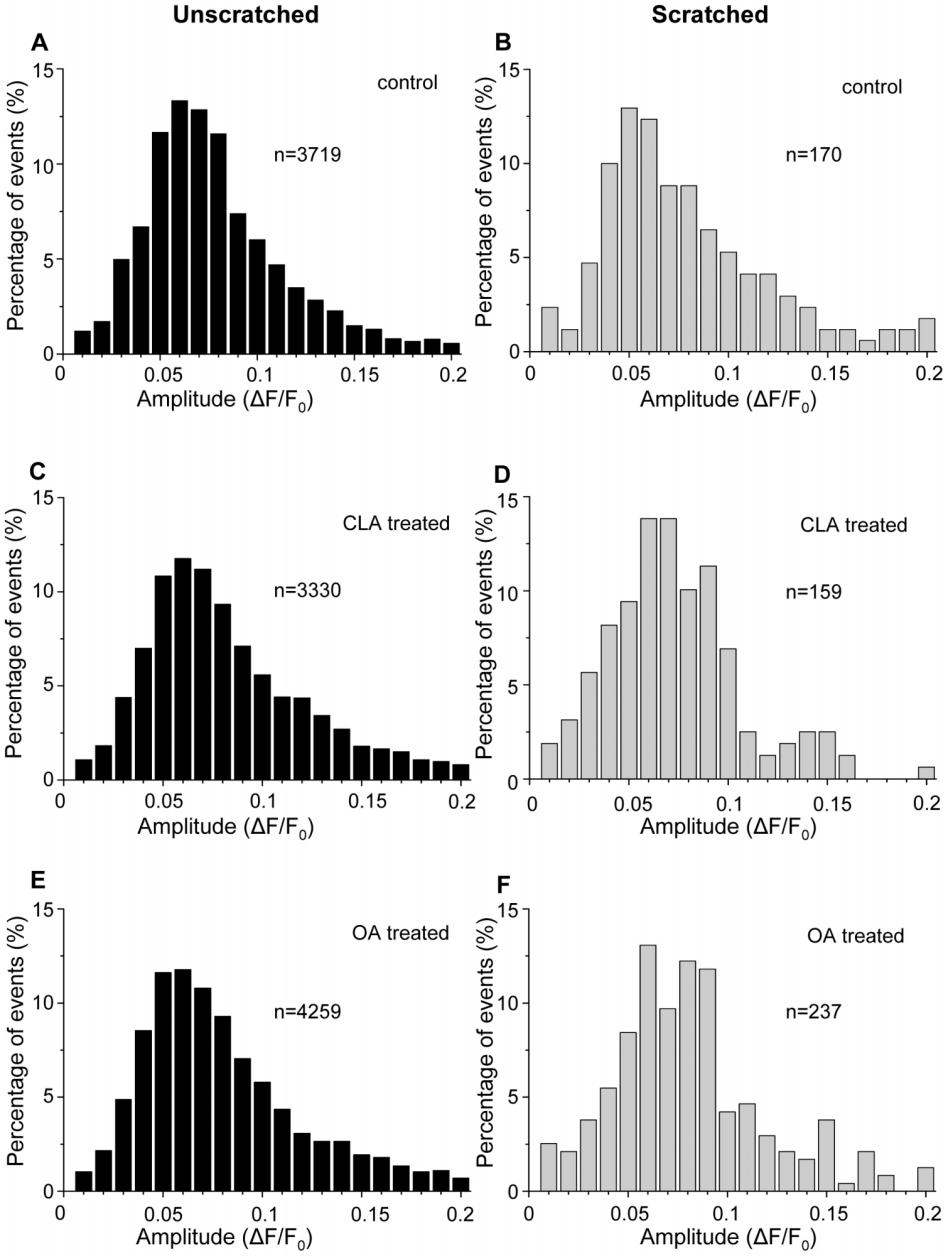
The amplitude of oscillations. Distribution histograms of the amplitude of oscillations in control (**A, B**), CLA treated (**C, D**) and OA treated (**E, F**) cultures measured by confocal microscopy. The cells were chosen either from unscratched coverslips (**A, C, E**) or from the cell groups next to the scratch in scratched coverslips (**B, C, F**). The “n” values give the number of Ca^2+^ transients (C: control, CLA: calyculin A, OA: okadaic acid).

In unscratched cultures the amplitudes of the spontaneous Ca^2+^ transients were significantly higher in the presence of phosphatase inhibitors than in control cultures (control: 0.082±0.001, CLA: 0.096±0.002, OA: 0.089±0.001), while no such increase was observable analyzing the data of cells next to the scratch (control: 0.093±0.008, CLA: 0.079±0.006, OA: 0.090±0.004). Furthermore, except for the CLA treated cells, the difference between the amplitude values measured in non-scratched cultures and in cells near the scratch was not significant (Fig. 3).

**Figure 4:**
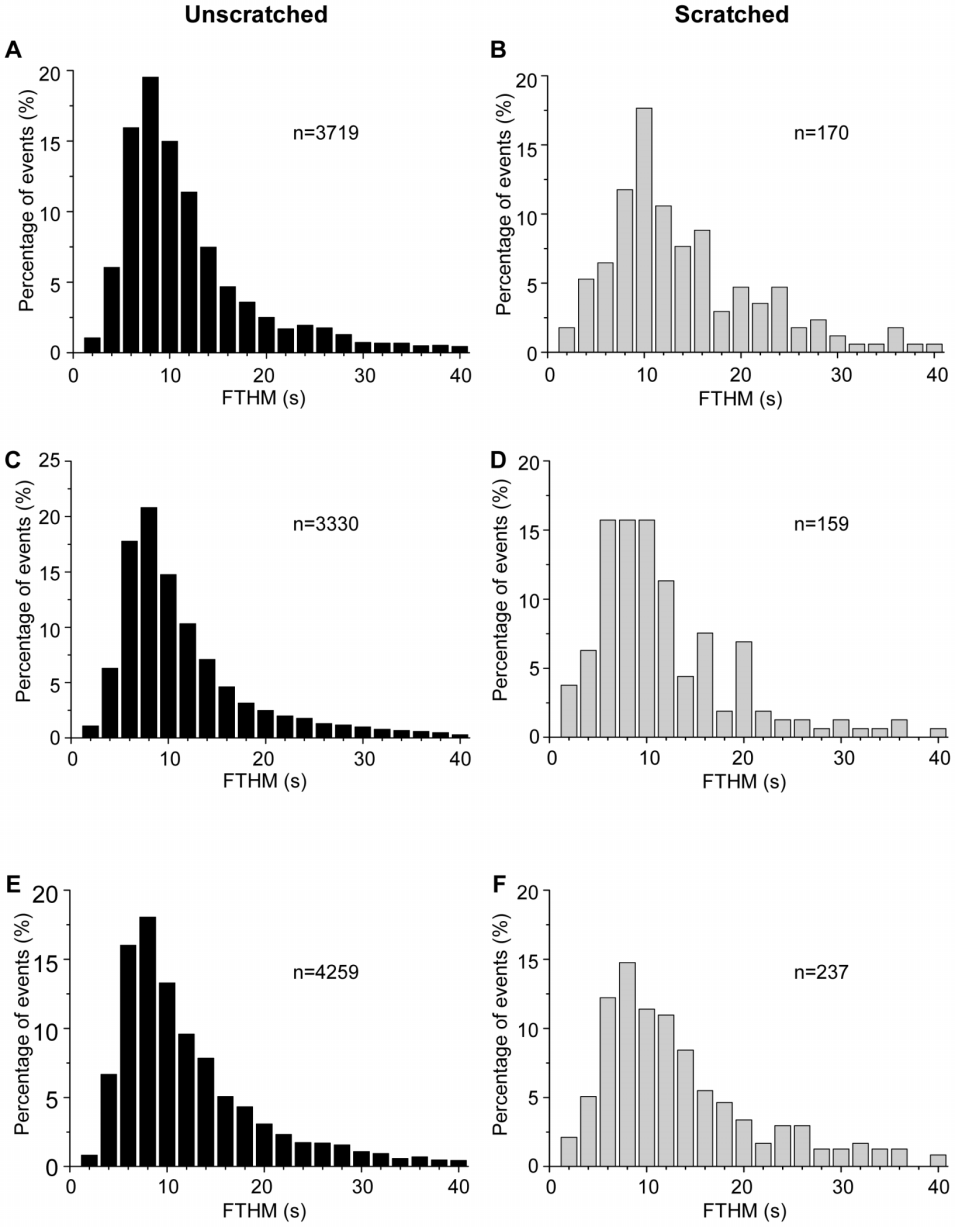
The Full time at half maximum (FTHM) values of oscillations. Distribution histograms of the Full time at half maximum (FTHM) values of oscillations in control (**A, B**), CLA treated (**C, D**) and OA treated (**E, F**) cultures measured by confocal microscopy. The cells were chosen either from unscratched coverslips (**A, C, E**) or from the cell groups next to the scratch in scratched coverslips (**B, C, F**). The “n” values give the number of Ca^2+^ transients. (C: control, CLA: calyculin A, OA, okadaic acid).

In control and in OA treated cultures the FTHM values were significantly higher in the vicinity of the scratch as compared to untouched areas (non-scratched control: 12.6±0.2 s, OA: 13.4±0.2 s; scratched control: 15.4±0.9 s, OA: 16.2±1.1 s). This difference was not statistically significant in case of CLA treated cells (non-scratched: 11.8±0.2 s, scratched: 11.5±0.6 s). In unscratched cultures CLA treatment decreased, while OA treatment increased the FTHM values. In cells near the scratch, the CLA treatment decreased significantly, while OA treatment did not change the FTHM values as compared to the control (Fig. 4).

The influence of the scratching on both parameters was parallel in control cultures, i.e. the amplitude as well as the FTHM increased. After the treatment by phosphatase inhibitors the measured values of these parameters (except for CLA treatment on FTHM) were also augmented.

### 2. Effects of the phosphatase inhibitors on keratinocyte cultures

**Figure 5:**
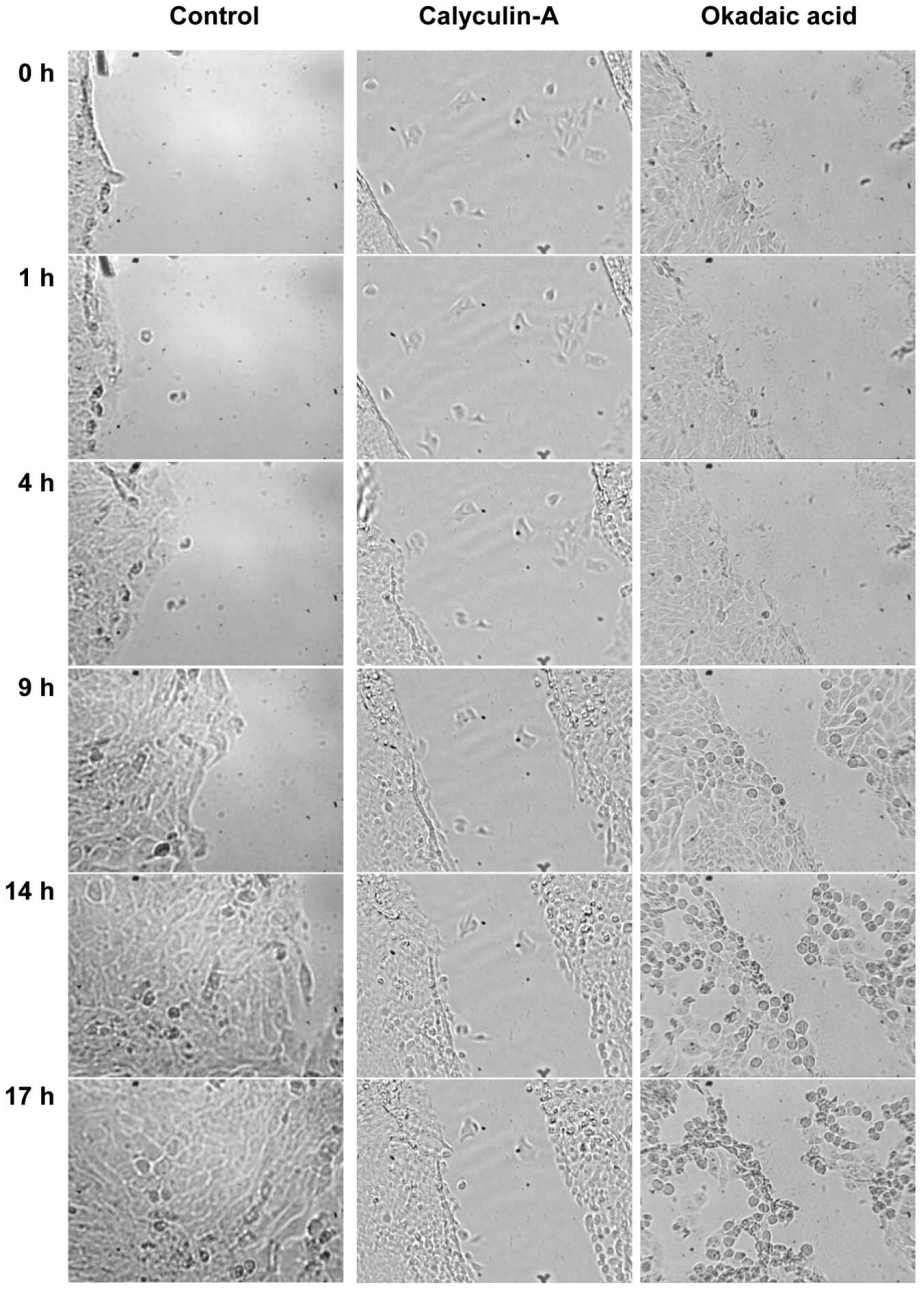
Time-lapse experiments. Representative images from time-lapse experiments of control (left panel), CLA treated (middle panel), and OA treated (right panel) cultures showing the altered migrating ability and wound healing as a consequence of phosphatase inhibitor treatment. Cultures were scratched with a 10 µl pipette tip and were placed on inverse microscopes in a 37°C incubator. Photographs were taken in every minute. Original magnification was 40×, 10×, and 25×, respectively.

Time-lapse experiments were carried out to demonstrate the migration of keratinocytes by examining control and phosphatase inhibitor treated cultures (Fig. 5). Control cells started to cover the scratched area (time-lapse video can be seen in Video S1), and they totally filled it in by the 17^th^ hour of the experiment. In contrast, CLA and OA treated cells were not able to cover the scratched area during the same time interval, and, furthermore, in case of OA treatment cells lost their extensions and their connections with their neighbors by the end of the experiment (Video S2). Similar morphological changes were not recognized on CLA treated cultures (Video S3).

### 3. Effect of OA and CLA on the migration of HaCaT cells

**Figure 6:**
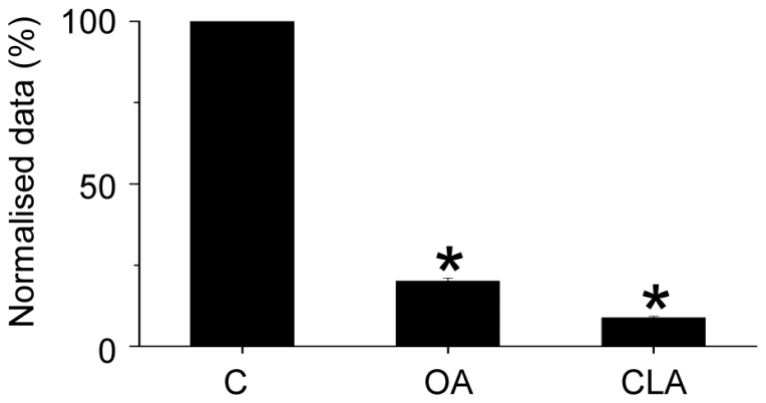
Effect of OA and CLA on the migration of HaCaT cells. Migration of HaCaT cells was assayed in a Quantitative Cell Migration Assay as described in the Materials and Methods at the indicated concentrations of OA and CLA. The optical density of stained cells migrated in the absence of OA and CLA was taken as 100%. Values represent mean ± SEM (n = 3).

To quantitatively assess the migration properties of the cells, they were incubated under low-serum conditions for 18 hours in the absence or presence of OA and CLA in the Boyden Chamber. Migration through the porous membrane to the other side was assessed by measuring optical density of the lysed cells stained with the dye provided in the migration assay kit. OA in 50 nM concentration decreased the chemoattractant-induced cell migration by about 80%. Similarly, 10 nM CLA also had a massive inhibitory effect on cell migration decreasing the normalized cell migration by 90% (Fig. 6).

## Discussion

Scratching a confluent layer of keratinocytes and subsequent filling of the wound bed with new cells represent a wound healing model often applied to study the mechanism of wound closure and restoration of barrier function of this cell type. Protein kinase and phosphatase enzymes together with the changes in [Ca^2+^]_i_ have been shown to possess a significant role in the regulation of cell migration and wound healing. The latter is especially important in case of the skin which is the first defense line of the body. Despite of the numerous studies there still is no clear consensus whether changes in [Ca^2+^]_i_ and phosphatase activities have parallel or antagonistic roles.

Our present results show that in cells from scratched regions the frequency of Ca^2+^-oscillations is significantly decreased compared to the cells from the untouched areas and the ratio of oscillating cells is also reduced. The characteristic parameters of these oscillations (amplitude, FTHM), however, were significantly higher in the scratched area. These observations suggest that the Ca^2+^ release processes in cells next to the scratch are less frequent but last longer and result in a greater change in [Ca^2+^]_i_ as compared with untouched cells. Other cell types like Cajal [24] and extraocular muscle cells [25] also show spontaneous calcium elevations, which are similar to those observed here on HaCaT cells considering both their amplitude and their time course.

On the other hand, enhancing the phosphorylation level of proteins by inhibition of Ser/Thr specific protein phosphatases with cell-permeable CLA and OA increased resting [Ca^2+^]_i_ and the frequency of Ca^2+^-oscillations in cells of both unscratched and scratched areas, however, cells close to the scratch still exhibited fewer number of oscillations than the unscratched ones. This [Ca^2+^]_i_ increasing effect of phosphatase inhibitors is in accordance with previous results suggesting that phosphatase inhibition may raise [Ca^2+^]_i_ and Ca^2+^ entry via enhancing the phosphorylation level of proteins involved in Ca^2+^-transport such as phospholamban, ryanodine receptor and plasma membrane Ca^2+^ channel [2], [16]. In non-scratched cultures the characteristic parameters of the Ca^2+^-transients (amplitude, FTHM) were calculated to be higher after the treatment by CLA and OA (except for FTHM in case of CLA treatment). These parameters showed remarkable alteration after scratching on phosphatase inhibitors treated cell cultures. Comparing the values measured on non-scratched cultures to the parameters obtained in cells next to the scratch, a synergistic relationship between phosphatases and the scratch-induced changes in [Ca^2+^]_i_ could be excluded. The magnitude of the scratch-induced [Ca^2+^]_i_ elevation was not significantly potentiated either by CLA or by OA treatment. The changes in the examined parameters observed in control cultures after scratching were not amplified by the application of phosphatase inhibitors rather they were diminished or vanished, however the application of phosphatase inhibitors on non-scratched cells had similar effect on the measured parameters as the scratch did.

Still standing key question is how changes in [Ca^2+^]_i_ and the phosphorylation state of certain proteins may contribute to the proliferation and migration of the cells during the wound healing process. According to the current literature this issue is controversial. On the one hand a burst increase in [Ca^2+^]_i_ was reported to stop directional movement of keratinocytes [12] and an increased phosphorylation level of myosin II together with an increased stress fiber formation resulted in a decreased hepatic cell migration [10]. On the other hand, protein-serine/threonine kinase inhibitors enhance the formation and extension of lamellipodia, a process believed to mark the start of events that lead to the migration of keratinocytes and wound healing [9]. In addition, inhibition of PP2A by 10 nM okadaic acid resulted in an increased extent of migration [11].

Movement of cells requires remodeling of the actin-cytoskeleton that includes formation of actin-myosin stress fibers via the Ca^2+^-dependent phosphorylation of the 20 kDa light chain of myosin II (MLC20) and RhoA/Rho-kinase induced phosphorylation and inactivation of myosin phosphatase [26]. It was shown that following wounding a rapid phosphorylation of MLC20 occurred as a prelude of cell polarization and migration which required a cytosolic Ca^2+^ flux and upstream activation of the p38/MAPK [27]. This early and rapid phosphorylation of MLC20 resulted in translocation of myosin IIA to the cell cortex primarily and this actin-myosin interaction was implicated in the membrane repair of wounded cells. In addition, RhoA/Rho-kinase was found to be essential for contraction and directed migration of keratinocytes [28]. Accordingly, inhibition of the protein phosphatases by CLA or OA would be expected to favor cell migration via increasing [Ca^2+^]_i_ as well as promoting the phosphorylation of both MLC20 and the inhibitory phosphorylation of the myosin phosphatase regulatory subunit as shown earlier [10], [29]. Nevertheless, CLA and OA were shown here to apparently halt migration of keratinocytes in both the wound healing assay and in the transwell migration assay. Myosin phosphorylation plays important roles in the cell attachment-detachment process especially during retraction of the tail which requires the force exerted by the actin-myosin stress fibers [30]. As cell migration involves repeated attachment-detachment processes it should be coupled with cyclic phosphorylation-dephosphorylation of MLC20. Our findings support the idea that phosphatase inhibitors cause a maintained phosphorylation of MLC20 with long-lasting stress fibers which eventually suppresses migration.

With respect to these findings the decrease of Ca^2+^-oscillations in cells close to the scratch may help keratinocytes to move into the gap between the cells caused by scratching. On the other hand lowering [Ca^2+^]_i_ may also be advantageous for the proliferation of keratinocytes since this process could be inhibited by increasing influx of Ca^2+^ into the cells [31].

## Supporting Information

Video S1 **Migration of keratinocytes in control culture.** Time-lapse video. Control cells started to cover the scratched area, and they totally filled it in by the 17^th^ hour of the experiment.
(MP4)

Video S2 **Migration of keratinocytes in ocadaic acid treated culture.** Time-lapse video. OA treated cells were not able to cover the scratched area and, furthermore, these cells lost their extensions and their connections with their neighbors by the end of the experiment.
(MP4)

Video S3 **Migration of keratinocytes in calyculin A treated culture.** Time-lapse video. CLA treated cells were not able to cover the scratched area, however, morphological changes similar to the OA treated cells were not recognized on CLA treated cultures.
(MP4)
